# *Revista de Saúde Pública* in scientific publications on Violence and Health (1967-2015)

**DOI:** 10.1590/S1518-8787.2016050000086

**Published:** 2016-10-26

**Authors:** Lilia Blima Schraiber, Claudia Barros, Ana Flávia Pires Lucas d’Oliveira, Maria Fernanda Tourinho Peres

**Affiliations:** I Departamento de Medicina Preventiva. Faculdade de Medicina. Universidade de São Paulo. São Paulo, SP, Brasil; II Programa de Pós-Graduação em Saúde Coletiva. Universidade Católica de Santos. Santos, SP, Brasil

**Keywords:** Violence, Public Health, Periodicals as Topic, history, Scientific Communication and Diffusion, Review, Historical Article

## Abstract

This article retrieved the publications from the *Revista de Saúde Pública* journal (from 1967 to 2015) on violence and health, on the SciELO and PubMed bases, by searching for the terms “violence”, “suicide”, “aggression”, “bullying”, and “external causes”, registered in any part of the text. We found 130 articles (the first one published in 1974). We observed: increase of publications over time, with decrease in the last five years; similar production volume in lethal and non-lethal violence; later publication of the latter; few studies in qualitative research; mostly descriptive production; and visualization of the problem more by the acts than by contexts or motivations and aggressors. Social markers were little approached, appearing, from largest to smallest frequency, social class, gender, race/ethnicity, and generation. Human rights were little used and only recently used as analytical framework, connected more to gender than to social class. Although *Revista de Saúde Pública* has registered the theme in its publications, consolidating it as scientific production line, there is still great explanatory theoretical rarefaction and little intersectionality between violence, social inequalities, and human rights.

## RESUMO

A produção da Revista de Saúde Pública (de 1967 até 2015) sobre violência e saúde foi recuperada nas bases SciELO e PubMed utilizando-se os termos “violência”, “suicídio”, “agressões”, “*bullying*” e “causas externas”, registrados em qualquer parte do texto. Foram encontrados 130 artigos (o primeiro deles publicado em 1974). Constatou-se: aumento das publicações no tempo, com decréscimo nos últimos cinco anos; volume similar de produção em violência letal e não letal; publicação mais tardia desta última; poucos estudos em pesquisa qualitativa; produção majoritariamente descritiva; e visibilização do problema, mais pelos atos que pelos contextos ou motivações e agressores. Os marcadores sociais foram pouco tematizados, aparecendo da maior para a menor frequência, classe social, gênero, raça/etnia e geração. Direitos humanos foi pouco, e apenas recentemente, usado como referencial analítico, conectado mais ao gênero do que à classe social. Embora a Revista de Saúde Pública tenha inscrito o tema em suas publicações, consolidando-o como linha de produção científica, há, ainda, grande rarefação teórica explicativa e pouca interseccionalidade entre violência, desigualdades sociais e direitos humanos.

## INTRODUCTION

Violence has only been recognized as a health issue about 20 years ago, and because of this recent history, it cannot be affirmed as a traditional theme. Because of the difficulties as an object of study, it has been characterized by diversity of research designs and plurality of definitions. Heise and Garcia-Moreno^9^, commenting on the difficulties in comparing data about intimate partner violence, show a number of variations in sampling designs, positive case definition, and selection of individuals to be researched; additionally, the authors also observe diversity produced in different sources of data, which applies to researches based on secondary data, a usual situation in studies on mortality by violence. Moreover, addressing violence in studies that produce primary data implies, in field work, ethical peculiarities, ranging from informed consent to the safety of respondents and researchers, including the training of the latter and reception to cases at risk of dying and others that demand health-care orientations^13^ ^-^ ^16^.

The *Revista de Saúde Pública* (RSP) journal, in its 50 years of existence, brings its collaboration both in the sense of recognition of this subject, consolidating a progressive presence of the production on violence among its publications, and bringing to the public the wide variety of studies that constitutes it. This article aimed to describe this production, interpreting it in the light of key issues, such as human rights violations and social inequalities.

## METHODS

To contextualize the production of RSP, we collected, from 1967 to 2015, by Pubmed and SciELO, the world and Brazilian production of six other journals of Public Health: *Cadernos de Saúde Pública* (CSP), *Ciência e Saúde Coletiva* (CSC), *Saúde e Sociedade* (S&Soc), *Physis: Revista de Saúde Coletiva* (Physis), *Interface – Comunicação, Saúde, e Educação* (Interface), and *Revista Brasileira de Epidemiologia* (RBE). We used the terms, in Portuguese, for: “violence”, “homicides”, “suicides”, “aggression”, “bullying”, and “external causes”, registered in any part of the article. However, we focused the study on the specific content of RSP publications.

Two forms of analysis of this production were performed: descriptive and analytical-interpretive. The description sought to characterize it according to 16 relevant aspects^2^ ^,^ ^4^ ^,^ ^7^ ^,^ ^8^ ^,^ ^10^ ^,^ ^12^ ^,^ ^14^ ^,^ ^15^ ^,^ ^18^, operated as categories of classification and analysis of the articles: “types of violence” studied; “year of publication”; “institution of origin” of the studies; “authors’ sex”; “methodological design”; “general approach of the study”; “data source”; “target population”; “discrimination of acts” committed; “identification of aggressors”, “context” and “motivations” of the violence; types of “examination of results”; “use of the term violence” in the study; and insertion of the referential of “social markers of difference” and of “human rights” in the study.

For the classification of the studies, we considered, at first, the traditional distinction of harms to health between deaths and non-fatal events, by adding internal progressive ratings to each group, in accordance with the literature on violence^5^ ^-^ ^10^.

Regarding temporality, the studies were grouped by five-year periods. The discrimination by “authors’ sex” considered that violence against women could motivate female authorship. We verified, in “methodological design”, if the studies approached their objects by qualitative or quantitative research, either in epidemiology, social sciences, and humanities or policies, planning, and management in health. In the “general approach of the study”, we considered if there was a more theoretical or empirical approach to the object, and in “data source”, if the study used primary or secondary data. The “target population” distinguished the researched age group and if it was by population surveys or access to schools, health services, or other institutions.

In “discrimination of the acts”, we verified if possible actions, such as slapping, beatings, humiliations, forced sex etc., were mentioned^9^, which determine the physical, psychological, and sexual natures of violences^5^. This was followed by the presence and discrimination of “aggressors”, characteristics of the “context” and “motivations” of the occurrences. In the “analysis of results”, we considered whether the approach was descriptive or analytical.

Finally, for the insertion of the referential of “social markers of difference” and of “human rights”, we considered mentions to and forms of use of “social class, gender, race/ethnicity, generation”^3^ ^,^ ^12^ and “rights”^15^, in the form of violations or affirmations of social, sexual, reproductive rights or of human dignity in general.

In the examination of analytical-interpretative articles, we verified if the studies presented a theoretical reflection, producing comprehensive summaries to explain their objects, under two different perspectives: understanding of the meanings attributed to the word “violence” and contributions to the explanatory densification of the scientific theories in our field.

Regarding the meaning of violence, first we considered the existence of a formal definition. Then, we inquired what empirical aspects were thus named, by examining whether the event was analyzed by articulation between acts, perpetrators, contexts, and motivations of the episodes, giving a more complete interpretation to violence as occurrence or event, or whether only some of those elements that make up violence were considered, giving it a partial meaning.

Regarding the explanatory densification, taking as guide of analysis the distinction between “idea”, as a first representation of reality, and “concept”, as a comprehensive theoretical formulation of reality^11^, we analyzed if “violence” has been used more abstractly as an idea or if it was an event approached socioculturally in connection to health. We based such procedure in the literature that points the polysemy of “violence”^14^ ^,^ ^15^ and a “theoretical rarefaction”, as little conceptual development in the studies, in many production chains of Public Health^2^ ^,^ ^17^. This rarefaction is often expressed by the larger endorsement to the ethic and political perspective of questions brought by social movements than to the conceptual formulation implied^1^.

Thus, we aimed to analyze how and whether the theoretical rarefaction was present in the production examined, considering if violence was fully approached and if it was analyzed as social and cultural action, to gain explanations that must be of social and anthropological, legal, and ethical nature, in view of being a social phenomenon and that is also target of legal standards. We verified if, in the analysis of violence, the markers of social difference and human rights were included, because, for these or those, the use may be only a mention, as background context of the studied case, without necessarily constituting an analytical reference for their understanding.

Finally, methodologically, it is worth noting that, by the qualitative nature of the conducted analysis, we follow the guideline of presenting the study results related to their interpretations^6^.

## RESULTS AND DISCUSSION

We found, for the world production, a total of 3,849 articles and, for the Brazilian production, 945, with 349 in CSC, 259 in CSP, 130 in RSP, 85 in S&Soc, 50 in Interface, 45 in RBE, and 27 in Physis.

This production of RSP was classified into two large groups: lethal and non-lethal violence^5^, adding the distinction of the family context of the event^8^ ^,^ ^9^, the community context^5^, and the institutional context of care^7^. We emphasized, as usual^10^, the sexual violence.

In lethal violence are: “external causes”, with studies that take all causes thus considered; “homicides”, “suicides”, and “accidents”, with specific studies for each of them. In non-lethal violence, there are the subgroups: “domestic”, involving situations with family members of any age; “institutional”, considering health-care situations between professionals and users; “community”, with studies of interpersonal violence in public environments such as schools, work; and “sexual”, with so-called studies and in which sexual violence was not part of the set of situations explored as family violence.

The studies were distributed in close volumes in the groups of lethal and non-lethal violence. In the first, “external causes” formed the largest sub-group and, in the second one, this corresponded to domestic violence against women (Table 1).

**Table 1:** Absolute and relative frequencies of the articles according to type of violence (1967-2015).

Types of violence	n	%
Lethal	64	100
External causes	32	50.0
Homicides	16	25.0
Accidents	9	14.1
Suicides	7	10.9
Non-lethal	66	100
Domestic violence		
Against women	35	53.0
Against children	5	7.6
Against adolescents	3	4.6
Against older adults	1	1.5
Community	13	19.7
Sexual	7	10.6
Institutional	2	3.0

**Figure 1:**
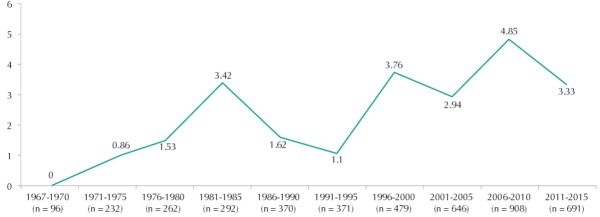
Temporal distribution of relative frequency of the articles on violence of any type in relation to the total number of articles published in the *Revista de Saúde Pública*, from 1967 to 2015.

**Figure 2:**
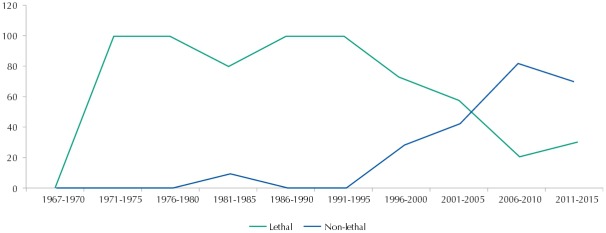
Temporal distribution of relative frequency of publication on lethal and non-lethal violence in relation to the articles on violence published in the *Revista de Saúde Pública*, from 1967 to 2015.

In terms of temporal trend, the topic of violence began to be published in the second five-year period, growing over the researched period and reaching its maximum rate, of 7.3%, in 2009 (Figure 1). In the last five years, however, the growth seems to have stopped, dropping its rate to 3.3% in the production of the Revista. In Figure 2, we can observe the later entrance of studies on non-lethal violence, which occupy the largest proportion of production from 2005 on.

Next, we examine lethal and non-lethal violences, according to the mentioned categories, part of them in Tables 2 and 3.

**Table 2:** Absolute and relative frequencies of the characteristics of articles about lethal violence (1967-2015).

Characteristic of the study – Lethal violence (n = 64)	n	%
Authors’ sex		
Male only	7	10.9
Female only	26	40.6
Both	29	45.3
Institutional authorship	2	3.1
Number of authors		
1-2	30	46.9
3-6	32	50.0
≥ 7	2	3.1
General approach		
Empirical	61	95.3
Technical or review	3	4.7
Instrument validation	0	0
Method		
Qualitative	0	0
Quantitative	64	100
Both	0	0
Examination of results		
Descriptive	43	67.2
Analytical	21	32.8

**Table 3:** Absolute and relative frequencies of the characteristics of articles on non-lethal violence (1967-2015).

Characteristic of the study – Non-lethal violence (n = 66)	n	%
Authors’ sex		
Male only	2	3.0
Female only	22	33.3
Both	42	63.6
Number of authors		
1-2	14	21.2
3-6	45	68.2
≥ 7	7	10.6
General approach		
Empirical	57	86.4
Technical or review	3	4.6
Questionnaire validation	6	9.1
Method		
Qualitative	10	15.2
Quantitative	55	84.9
Both	0	0
Examination of results		
Descriptive	10	15.2
Analytic	56	84.9

### Lethal Violence

The first article of this group was about suicide, in 1974 (v.8, n.1). The first paper on “external causes” was published in 1976 (v.10, n.1) and the first on “homicides”, only in 1996 (v.31, n.1). Suicide appears to have been the first question to raise the researchers’ interest; however, there was no reference to the term violence, which occurred only in 2010, unlike studies on “external causes”, which mention the term since their beginning. Whilst about “accidents”, their first study is from 1975 (v.9, n.3) and neither mentioned “violence”.

On the set of this production in lethal violence, there was a change in the way of approaching violence. Until the mid-1990s, the term emerges in articles not as a defined object of study, but as a circumstance of deaths, i.e., “violent” deaths. Often, the term referred only to homicides and suicides; in other times, it included accidents and poisonings. We observed oscillation in the use of the term, which, however, was used mostly as a synonym of external causes.

The mentioned article about homicides, from 1996 (v.31, n.1), can be considered a transition, highlighting violence already in the title. Also, in the discussion, it mentioned the relation of its growth and male mortality excess with the structural social conditions. But it did not present a gender analysis about this difference in risk of death between men and women, although the difference has been recognized very early. The first publication to explore homicide in the female population occurred only in 2011 (v.45, n.3), terming it “femicide” and treating it as a gender issue.

Regarding authors’ sex, women predominated in all subgroups of studies on lethal violence, either as an overall proportion of women out of the total of authorships, or as exclusive women authorships (Table 2). In its largest subgroup, “external causes”, women represented two thirds of researchers. The institutions were mostly located in the Southeast region (90.0%), and we also analyzed a study in Spanish, from Mexico (1996, v.31, n.1).

The studies used basically secondary data. Descriptive studies predominated. Among the analytical ones, ecological studies stood out. But, even within the descriptive line, there was: in 2005, examination of the role of firearms in homicides; in 2010 and 2011, examination of the mortality excess of black individuals; and in 2014, in the geopolitical distribution of homicides, identification of the non-random form of spatialization of those deaths in unequal sociogeographical areas.

The data source was, for all, the declaration of death, by the highest temporal scope, coverage, and quality of data. However, this results in important gaps for the study of violence. Some have explored certain information from contexts, but the mostly examined feature were the acts performed. We do not know anything about the relationship between victim and aggressor, who are they and what are the circumstances and motivations. In this sense, the studies did not gather data necessary for the articulation of the internal elements of violence, thus preventing a more comprehensive analysis of them as social and health facts. The use of violence, therefore, had, in these early studies, important symbolic meaning, more than operated as explanatory reference in the examination of data. Thereby, without analyzing violence, the deaths caused by it neither could have their reasons explained, thus the more descriptive nature of these studies. On the other hand, they were important for the construction of the discourse that introduced and consolidated violence as a health issue in Brazil.

The studies, as a whole, considered the sociodemographic variables “sex”, “age”, “skin color”, “income”, or “schooling”, used, however, as descriptors of social characteristics. Therefore, the terms social class, gender, and race did not represent analyses from the perspective of social or cultural difference and of ethical-political inequalities involved. Social class was treated, mostly, by the social condition of poverty, emptying the concept toward an only socioeconomic characteristic. A similar reduction also occurred regarding “gender”, in use restricted to the comparison of events between men and women, but without placing it in the sociocultural understanding that the differences represent, reiterating what was verified in the production of Public Health in general^1^.

However, an article from 2009 (v.43, n.1), on external causes, and another from 2013 (v.47, n.2), about maternal mortality, were the first that, when mentioning social class and gender, sought some sociocultural interpretation, in addition to being the only ones to use the referential of human rights, not observed in any of the studies on homicides. The discussion of violence connected to rights, as violation or, in its prevention, as promotion of these rights, is virtually absent. The same was found in the connection between violence and various social markers of differences, which were little analyzed, although mentioned. Efforts in this direction constitute studies from 2001 (v.35, n.6) and 2005 (v.39, n.1), in approaching social inequities in the distribution of risk of homicide, in addition to a study from 2011 (v.45, n.4), whose object was the differential of death by race/skin color, and the mentioned study from 2011 (v.45, n.3) on femicide, which better used gender as an analytical framework.

These latest indications point to a recent trend in the studies regarding the more analytical and explanatory concern.

### Non-Lethal Violence

In the context of “domestic violence”, the largest segment was composed of studies on women, which, from 35 articles, had 86.0% addressing intimate partners. At the other end, there was only one article on older adults, the less studied population group.

Epidemiological studies were most, which, together with lethal violence, prevailed epidemiological researches, reflecting the vocation of RSP. But, unlike “lethal violence”, in this group most studies developed analyses of their data: 31 were cross-sectional studies (20 in services and 11 in population), one of case-control, and two, cohort.

Regarding authors’ sex, we did not observe polarization for females (Table 3), and, taking in particular the studies of violence against women, this was also not found, keeping the ratio between male and female authorship found in lethal violence. The greater interest expected in the subject of domestic violence by women researchers, therefore, was not verified.

The studies came mainly from the Southeast region, although in much smaller proportion (60.0%) than in the case of lethal violence. We observed four studies in Spanish, from Mexico, one coauthored with the United States and two with Brazilian and North American joint authorship, totaling a higher number than in lethal violence.

Violence appeared named and approached as a central issue of study on most articles, marking an important difference with the lethal violence studies. However, the conceptualization of the term remained low. Few studies defined violence and, when they did, they leaned on the World Health Organization^10^ or in international conferences of rights for women. Nevertheless, the definition, in general registered in the introduction of the articles, rarely articulated to the discussion of the findings.

The first study on domestic violence is from 1998 (v.32, n.5), approaching, however, the validation of three measurement instruments, only one of them related to violence. The first paper on violence against children was published in 1999 (v.33, n.6). The first publication with primary data on violence against women was from 2002 (v.36, n.4), and against adolescents, from 2005 (v.39, n.5).

Several terms were used to record violence against women, and “conjugal”, “domestic”, and “familiar” were the most frequent ones. In the ones about children, there were also several nominations: “abuse”, “corporal punishment”, “negligence”, and “mistreatment”. Even dealing with family violence, in the studies on children and adolescents the samples were in schools or in services. In the case of studies on women, in addition to the research in services, household surveys also took place.

On violence against women, there were three related and extensively studied topics: the context of pregnancy; the use of alcohol and drugs; and depression and mental suffering. This highlights the examination of the consequences for health.

But the problematic valued was the invisibility of violence on health. Treated explicitly as such, it expressed itself by the effort to meet prevalences and risk factors. In some studies, perpetrators and their relationship with the victim, in contexts of alcohol and drugs use, were also explored. The acts, such as in lethal violence, were widely explored, but the severity of episodes or overlays of physical, psychological, or sexual violence were not systematically examined. The perceptions about violence or additional contexts to the abuse of alcohol and drugs or pregnancy were barely addressed.

In the case of sexual violence, the first article was from 2003 (v.37, n.1). Totaling seven studies, four were on teenagers, one of them adding children, two on women, and one exclusively on professionals. Of the seven, three focused on the care to the victims and three of the four studies with adolescents were carried out in schools. If we add to them studies on family violence, schools predominate as spaces of research on adolescents.

Regarding the markers of social difference, “gender” was cited by most articles on violence against women (n = 23), but it worked partially as a category of analysis, again reiterating what was found for Public Health in general^1^. In the studies on sexual violence, this marker appeared only in two of the seven studies, with two studies on women that not even mentioned it. “Gender” was also not present in studies on children and adolescents, and when the parents’ behavior was approached, the woman was not treated as a specific condition of gender. Additionally, the very parenting was not discussed, not even presented, from the generational perspective.

Skin color had low mention in the demographic characterizations and its use to discuss racial inequality was almost null. Socioeconomic inequality, as in lethal violence, showed no significant discussion about social class. In some of these articles, the authors observed connection with gender, and one article from 2000 refers to generation as its central theme, addressing children. This was the only article that approached this social marker. Thus, in relation to the lethal violence studies, we can say that the non-lethal studies used a little more of social markers as analytical categories.

The reference to human rights appeared in 12 articles: one on violence against children, another against adolescents, and 10 against women. Generally, the reference was used defining violence as a violation of rights, but this inscription was restricted to the introduction of the articles, with less impact on the discussion.

Examining “institutional violence”, the only two existing studies were of qualitative research and approached health policy and management of services. One, from 1984 (v.18, n.2), used documents, addressing the sterilization of women as abuse and coercion, without discriminating acts or aggressors. The other, from 2009 (v.43, Suppl.1), focused on psychiatric and mental health services, using interviews and focus group.

Two women and one man totaled the researchers of institutional violence, with one study in the Southeast region (v.18, n.2) and another in the Northeast (v.43, Suppl.1). Both discussed human rights, but only the 1984 study (v.18, n.2) that referred to women also addressed “social class” and “race” articulately with “gender”, in an analysis of the reproductive rights of women.

Finally, on “community violence”, the first article was from 1999 (v.33, n.2), approaching physical aggression and “social class”, in case-control study, with people hospitalized in emergency room. Of the 13 existing articles, three were qualitative researches, one a review, and two used focus groups, all analytical. The remaining 10 were epidemiological studies: one, ecological study with secondary data, and nine with primary data. Of the latter, four were in schools; two in emergency room; one in health services; one with drivers and collectors, and another by population survey. Aside from the ecological study, the transverse studies predominated.

In community violence, the ratio between men and women researchers remained, in the proportion of 70.0% of men and 75.0% of women, and the predominance of the Southeast region also remained between the institutions. Violence was defined as physical aggression, mistreatment, rape, harassment, insults, or intimidation, but there was no formal definition, as it was not used conceptually, although the given meanings effectively constitute acts identified to the concept^15^.

The most frequent mention of race/color occurred in community violence, also with reference to the markers “social class” and “gender”. However, the use still remains more descriptive than analytical. The reference to human rights appeared only in two studies: the mentioned ecological one, from 2009, dealing with police violence, and the review study, from 2006.

### Final Considerations

The studies on lethal violence have inserted the theme in our environment; however, those on non-lethal violence were the ones that most approached violence as an issue.

Temporally, there was, for all, changes in approaching violence, articulating it increasingly with social and human rights markers. Of these markers, socioeconomic inequality was clearly the most approached one, followed by inequalities of gender, race, and, last, generation. This is also due to the fact that violence itself as event of life in society failed to have its examination completed, because, although almost all studies cared about showing the acts of violence, the empirical positivity of a possible concept remained restricted to them. And if the acts tell to health something about the severity of violence and the ways of its realization, this restriction leaves out the intention of the agent of this act and all the social context that generates such intentionality. Conceptualizing violence demands this joint understanding between acts, contexts, and intentions to approach the behavior of social subject of one who engages in such acts in the life of public relations (in urban space or institutions) and private relations (family or friendship). The concept of violence as this intentional behavior, by the cultural stimulus in that direction, as in gender issues^16^, or by the economic and social inequalities, and not as a natural base behavior of human beings, is well characterized in Minayo^13^, author, paradoxically, much mentioned in these studies on violence.

Thus, the big issue addressed persists in the demonstration that violence is a health problem, seeming to be, until today, the motivation of the researches. In addition, these really distinct forms to visualize violence have in common the fact that, if the concern about social inequality, in the reference to poverty, was the first social realm to be referred to, human rights are the last, by the low frequency with which they are included in studies and for only appearing on the publications from 2005 on, articulating more to gender than to social class.

Historically, RSP has complied with the initial commitment of inscribing the topic of violence in our field, giving it visibility as an object of knowledge, but, undoubtedly, greater encouragement must be given to studies that go beyond such first approach toward more substantive analyses.
